# Perfluoroalkyl Chemicals and Male Reproductive Health: Do PFOA and PFOS Increase Risk for Male Infertility?

**DOI:** 10.3390/ijerph18073794

**Published:** 2021-04-05

**Authors:** Pheruza Tarapore, Bin Ouyang

**Affiliations:** 1 Department of Environmental and Public Health Sciences, University of Cincinnati Medical Center, Cincinnati, OH 45267, USA; bin.ouyang@uc.edu; 2 Center of Environmental Genetics, University of Cincinnati Medical Center, Cincinnati, OH 45267, USA; 3 Cincinnati Cancer Center, University of Cincinnati, Cincinnati, OH 45267, USA

**Keywords:** PFOA, PFOS, perfluorooctanoate, perfluorooctane sulfonate, testosterone, spermatogenesis, sperm, mice, rats, epidemiological

## Abstract

Poly- and perfluoroalkyl substances (PFAS) are manmade synthetic chemicals which have been in existence for over 70 years. Though they are currently being phased out, their persistence in the environment is widespread. There is increasing evidence linking PFAS exposure to health effects, an issue of concern since PFAS such as perfluorooctane sulfonate (PFOS) and perfluorooctanoic acid (PFOA) bioaccumulate in humans, with a half-life of years. Many epidemiological studies suggest that, worldwide, semen quality has decreased over the past several decades. One of the most worrying effects of PFOS and PFOA is their associations with lower testosterone levels, similar to clinical observations in infertile men. This review thus focuses on PFOS/PFOA-associated effects on male reproductive health. The sources of PFAS in drinking water are listed. The current epidemiological studies linking increased exposure to PFAS with lowered testosterone and semen quality, and evidence from rodent studies supporting their function as endocrine disruptors on the reproductive system, exhibiting non-monotonic dose responses, are noted. Finally, their mechanisms of action and possible toxic effects on the Leydig, Sertoli, and germ cells are discussed. Future research efforts must consider utilizing better human model systems for exposure, using more accurate PFAS exposure susceptibility windows, and improvements in statistical modeling of data to account for the endocrine disruptor properties of PFAS.

## 1. Introduction

Poly- and perfluoroalkyl substances (PFAS) comprise more than 3000 individual compounds and generally contain a carbon chain backbone of 4 to 14 atoms in length and a charged functional moiety, such as a sulfonate or carboxylate [1]. Of all the long-chain PFAS family members, perfluorooctane sulfonate (PFOS) and perfluorooctanoic acid (PFOA) are the most studied PFAS. PFAS have been manufactured for over 70 years [1], and though they are currently being phased out in the US, their persistence in the environment is widespread and concerning to human health. Additional reasons for concern are reports of bioaccumulation of PFAS in humans and wildlife and increasing evidence linking environmentally relevant exposures to health effects. Both PFOS and PFOA have a substantial database of epidemiological, pharmacokinetic, toxicological, and mechanistic studies [2,3,4,5,6,7,8]. The chemical and thermal stability of a perfluoroalkyl moiety, and its hydrophobic and lipophobic nature, lead to its incorporation into surfactants, additives, and polymers by a variety of industries [9,10]. The carbon–fluorine (C-F) bond, being the strongest covalent bond, gives PFAS its thermal stability [11]. PFOS and PFOA have an eight-carbon-atom backbone. They have been used in an array of formulations, in firefighting foams, in hydraulic fluids for aircraft, and for waterproofing textiles and non-stick household cookware [1,12,13]. The present PFAS contamination within the environment is the result of consumer–product degradation, firefighting activities, and discharges from PFAS manufacturing facilities [14].

Epidemiological studies have shown that human PFOA and PFOS exposure is probably linked with increased risk for thyroid disease, high cholesterol, ulcerative colitis, kidney, testicular and prostate cancer, and pregnancy-induced hypertension [15,16,17]. In addition, PFAS has been found associated with adverse effects on the immune [18], endocrine, metabolic, and reproductive systems (including fertility and pregnancy outcomes) [19,20,21,22]. However, the weight of evidence supporting these associations varies by outcome, dose of PFAS, and the specific PFAS examined. Although many biological systems are adversely impacted by PFAS, this review focuses only on PFOS/PFOA-associated effects on male reproductive health, and how they may increase susceptibility to male infertility. Epidemiological studies suggest that semen quality has decreased over the past several decades in different countries throughout the world (reviewed in Gabrielsen and Tanrikut, 2016 [23]). The incidence of testicular cancer has also increased in the past 50 years [24,25]. Some studies have shown a strong association between male subfertility and subsequent risk of testicular cancer [26,27,28], suggesting common etiologic factors for infertility and testicular cancer.

One of the most worrying effects of PFOS/PFOA is their effect on Leydig cells in the rat testis, causing Leydig cell hyperplasia [29,30] and lower testosterone levels [31,32], similar to clinical observations in infertile men [33,34,35,36,37,38,39,40]. In our review, rodent studies are examined for common threads and possible endocrine disruptor activities of PFAS. Reports from animal and epidemiological studies implicate PFAS as an endocrine disruptor [15,34,36,41,42]. Endocrine disruptors are exogenous chemicals that can function as agonists or antagonists of hormones, thus disrupting hormone signaling or production [43]. Organs which depend on a constant influx of hormones for proper functioning, such as the testis and ovary, are especially vulnerable to the endocrine disruptor effects of PFAS, predominantly during windows of susceptibility, such as fetal development, infancy, puberty, and aging. Because of this, several epidemiological and rodent studies examine associations between neonatal and pubertal PFAS exposure and male reproductive health outcomes, as detailed later in this review. Early-life exposure to endocrine disruptors has been associated with epigenetic reprogramming, which manifests effects later in life, to increase the risk of disease [44,45]. In this context, we discuss the role of neonatal or pubertal exposures to environmental PFAS in increasing the incidence of reduced sperm counts [37,46] in adults.

In summary, this review discusses the various sources of exposure to PFOS/PFOA, leading to their high concentrations in the general population and in occupational groups. Levels of PFAS exposure in the developing fetus, and with the corresponding birth weight and size, is examined as a separate section, as gestational exposures have wide implications for increasing the risk of male infertility at adulthood. Evidence for sex differences resulting in differential PFOS/PFOA exposure in rodents and in humans is examined as a variable affecting the suitability of rodents as models for PFOS/PFOA exposures. The epidemiological and rodent studies are examined keeping in mind the non-monotonic (non-linear) dose response [47,48,49] usually exhibited by endocrine disruptors. Challenges, data-gaps, and areas for further research are addressed towards the end of each section.

## 2. Exposure Sources and Concentrations in the Population

PFAS are found in wastewater, as well as in rivers and lakes. They are present in drinking water, storm water, and groundwater [12,50,51,52,53,54,55]. The PFAS not removed in wastewater treatment plants and, consequently, released into the environment are a concern because of the possible presence of these compounds in water used for drinking and recreation. This existence of PFAS in drinking water is an alarming trend, since PFAS persist in humans, with a half-life of several years. Today, PFAS are ubiquitous in the serum of almost all US residents and in populations worldwide [56,57,58]. Continued exposure to even relatively low concentrations in drinking water can substantially increase total human exposure due to its bioaccumulation in serum.

Treated wastewater used for irrigation, or released into rivers, has been linked to PFAS in groundwater [59] and in rivers [60]. This appears to occur because conventional wastewater treatment plants are ineffective at PFAS degradation because of their highly stable covalent C-F bond, their low concentration in water, and their high hydrophilicity [12,61]. Biological treatment can break the C-C bond backbone, but this leads to the formation of short-chain PFAS. This may result in the concentration of PFAS being higher in treated water than in untreated water.

Wastewater facilities which incorporate a dual-filter design, carefully monitor for breakthrough, and frequently change filters have found that granular activated carbon filtration is highly effective at removing PFOA [57]. Using the West Virginia/Ohio Valley cohort, researchers have reported decreases in water PFOA from 1.9 to 4.9 ng/mL (parts per billion) to non-quantifiable (<0.016 ng/mL) or non-detectable levels [57] within 1 year. This was accompanied by a decrease in serum PFOA concentration (after water filtration) by ~26% per year, indicating a median serum PFOA half-life of 2.3 years [57]. This study contrasts with a study by Olsen et al. (2007) [62], who estimated that the geometric mean serum elimination half-life of PFOA was 3.8 (95% CI, 3.1–4.4) years for a group of 26 retired workers with previous occupational exposure. Results utilizing a German cohort of 354 men, women, and children showed declines from baseline serum PFOA concentrations of 10–20% per year after filtration of drinking water [54]. However, these apparent contradictions probably reflect differences in sources of drinking water, whether bottled, well-water, or from rivers [57,63], and possibly also additional sources of PFAS exposure inherent in lifestyle choices [55,64,65,66] which the population may favor. In fact, one of the more intriguing observations emerging from the West Virginia/Ohio Valley cohort study was the significant effect of homegrown vegetable consumption on the apparent half-life of PFOA [57].

The primary exposure source for the general population, apart from drinking water, is diet [55,64,65] and the inhalation of PFAS-contaminated indoor air and dust (from furnishings) [66]. PFAS has been detected in 100% of people tested [67,68,69,70], with the exposure of Americans to PFAS ranging from an average of 0.9 ng/mL to over 100 ng/mL. However, populations in Ronneby, Sweden, where up to one third of households were exposed to PFAS-contaminated drinking water, reportedly have PFOS and PFOA concentrations in their blood serum as high as 1500 ng/mL and 92 ng/mL, respectively [71]. In the US, West Virginia/Ohio Valley residents tested from 2005 to 2006 who drank PFAS-contaminated water close to a chemical plant had a mean level of serum PFOA and PFOS of 80 ng/mL (range 0.25–17,556.6 ng/mL) and 22 ng/mL (range 0.25–759.2 ng/mL), respectively [72]. This serum level did not distinguish between residents and factory workers.

Examples of PFAS-exposed occupational groups include the military and firefighters using flame-retardant foams [73,74], World Trade Center first responders [75], and PFAS manufacturing workers [63,76]. The PFAS factory workers appear to have the highest PFAS levels. Occupational exposure in factory workers has shown blood PFOA levels ranging from 2.25 μg/mL in DuPont workers in China [77] to around 100 μg/mL in 3M factory workers [36]. Olsen et al., 2007 [62] examined the serum of retired PFAS production workers and found mean serum levels of PFOS at 800 ng/mL (range, 145–3490 ng/mL) and of PFOA at 691 ng/mL (range, 72–5100 ng/mL). A recent study examining occupational exposure to PFAS and serum levels of PFOS and PFOA in an aging population (55–74 years, *n* = 154) from upstate New York [78] showed 25% higher PFOS and 80% higher PFOA levels in study participants compared to the National Health and Nutrition Examination Survey (NHANES). Among these, workers with high cumulative workplace exposure had 34% higher PFOS levels compared to the group without occupational exposure [78].

In 2016, the United States Environmental Protection Agency (US EPA) established drinking water Health Advisories of 70 ng/L (70 parts per trillion) for individual and total concentrations of PFOA and PFOS [79]. However, these are non-regulatory advisories and are presently deemed to be insufficiently protective. Current research focuses on the use of strong anion-exchange resins, which seem to remove both long- and short-chain PFAS. However, since their adsorption capacity for short-chain PFAS is lower than that for long-chain PFAS, the removal of short-chain PFAS is more difficult. Further, in the “real” world, there is usually organic matter mixed with the PFAS. Hence, studies on PFAS removal should incorporate experimental conditions which more closely mimic the environmental milieu. The rapid breakdown of PFAS into short-chain PFAS and the repeated regeneration of PFAS-exhausted adsorbents are other factors which require consideration and more research. Newer techniques such as advanced reduction processes also show promise in the removal of PFAS from water [80]. More details concerning research on the removal of PFAS from water by adsorption are reported in the literature [11].

## 3. Fetal Exposure to PFOS/PFOA Correlate with Changes in Birth Weight and Size

The very desirable chemical properties of PFAS are, ironically, responsible for their environmental persistence and long half-lives in humans. PFOA and PFOS accumulate in the serum, liver, lungs, brain, and kidney, in humans [81]; they are not easily metabolized, with half-life values of PFOS and PFOA in humans ranging from 2.3 to 5.4 years [54,57,62,82]. Studies in rats have shown PFAS accumulation in the testes [83,84] as well as these other organs.

Mice with gestational PFOA exposure show increased placenta weight, reduced fetal –placental weight ratios, and the presence of placental lesions, suggesting a role for placental toxicity [4,85]. Since PFAS can pass through the placenta to the fetus [86,87,88,89], exposure to PFAS starts from the womb. Infants are a particularly vulnerable subpopulation for PFOA’s developmental effects [4]. Their exposure from the mother drinking contaminated water during pregnancy, through breast milk after birth, and/or from formula prepared with contaminated drinking water has been reported [4,63,76,85,86,87,88,89,90]. In a cross-sectional study of singleton deliveries in the US, cord serum samples (*n* = 293) were analyzed for PFOS and PFOA. PFOA was detected in 100% and PFOS was detected in greater than 99% of cord blood serum samples [56]. Both PFOS and PFOA concentrations were found to be negatively associated with birth weight and size. However, a study utilizing the Danish National Birth Cohort with 1400 mother–infant pairs showed that only maternal plasma PFOA levels correlated with reduced birth weight [58]. A third study [91] incorporating data collected from 428 mother–infant pairs in Sapporo, Japan, indicated that in utero exposure to low levels of PFOS (measured from maternal serum) negatively correlated with birth weight in female infants only, with PFOA showing no such association. The inconsistencies in these three reports could be due to the timeframe of serum collection or the overall range of concentrations of PFOS/PFOA in the blood. For example, Fei et al. (2007) [58] reported that PFOS and PFOA levels in maternal plasma were, on average, 35.3 ng/mL (range, 6.4 to 106.7 ng/mL) and 5.6 ng/mL (range, <1.0 to 41.5 ng/mL), respectively. Apelberg et al. 2007 [56] reported that the median cord serum PFOS and PFOA levels were 5 ng/mL (range, <0.2 to 34.8 ng/mL) and 1.6 ng/mL (range, 0.3 to 7.1 ng/mL), respectively, while Washino, 2009 [91] showed average PFOS and PFOA levels in maternal serum to be 5.6 ng/mL (range, 1.3 to 16.2 ng/mL) and 1.4 ng/mL (range, <0.5 to 5.3 ng/mL), respectively.

These reports of embryonic exposure to PFOS and PFOA causing changes in birth body weight are a concern because these chemicals may not only directly disrupt fetal development of the male reproductive organs [32,92,93] but may have effects on steroidogenic hormone levels and sperm parameters at adulthood [31,32,46]. As outlined in the Barker Hypothesis [44,45] on the developmental origin of disease, an endocrine disruptor is capable of inducing epigenetic changes (discussed in modes of action) in different organ systems; some of these changes may remain hidden until adulthood, when they increase the risk for disease.

## 4. Studies Linking Environmentally Relevant Exposure to Male Reproductive Health

### 4.1. Sex Differences in Elimination Rates in Rats and Higher PFOS/PFOA Levels in Males in the Human General Population

In the rat, not only is the elimination half-life of PFOA shorter than that in monkeys and humans, but a notable sex difference exists [94,95,96,97,98], with females excreting the PFOA much more rapidly (half-life (t1/2) less than 1 day) than males (t1/2 = 15 days). One study found that testosterone affected the PFOA’s urinary elimination and was thus a key determinant of the sex difference in PFOA elimination in rats [99]. The transporter molecules (organic anion transporter) OAT2 and OAT3 were found to be responsible for PFOA transport in rat kidneys [99]. OAT2 mRNA levels in male rats were only 13% of that in female rats and were negatively regulated by testosterone and positively regulated by estrogen. This sex difference in the elimination of PFOA contrasts with the serum half-life of PFOA in mice, monkeys, and humans, which, while different, did not show sex-dependent differences in elimination (reviewed in Post et al., 2012 [100]). However, as noted by Olsen et al., 2008 [101] using the American Red Cross database, the geometric mean plasma concentration for PFOS was statistically significantly higher for males (17.1 ng/mL) than females (12.3 ng/mL). The geometric mean plasma concentration for PFOA in the general human population was 3.4 ng/mL (95% CI 3.3−3.6) but significantly higher for males (3.9 ng/mL) than females (3.0 ng/mL). Support for these sex-based differences have also been observed in the NHANES database [67]. This argues for a mechanism by which human males retain higher levels of PFOA and PFOS in their system. There is only one human study from Ronneby, Sweden, where a marked sex difference was noted with more rapid PFOS elimination in women but only marginally for PFOA [71]. The fact that the half-life of PFOA and PFOS in humans is much longer (measured in years) than in rodents (measured in hours or days), and the renal clearance in humans is negligible, contrary to the active excretion in animals, makes human risk assessment based on animal experiments problematic for these chemicals. However, the higher levels of PFAS in human and rat males than in females, as well as the ability to control for exogenous factors when performing animal studies, makes rodent studies essential to understand the mechanism underlying PFAS action in testes.

### 4.2. Epidemiological Evidence Linking PFOS and PFOA to Human Male Reproductive Health

The relation of PFAS to various fertility parameters, such as levels of reproductive hormones and sperm quality and levels, has been examined in many epidemiologic studies.

Epidemiological evidence shows that PFOA and PFAS exert significant effects on male reproduction parameters. Workers at the 3M company (Saint Paul, MN, USA), where PFOA was produced, had a 10% increase in mean estradiol levels observed among employees who had the highest levels of serum PFOA. However, this association was confounded by body mass index and became non-significant [102]. A cross-sectional study on 212 exposed males from the Veneto region, Italy, found that increased levels of PFOA (but not PFOS) in serum and seminal fluids positively correlated with circulating testosterone and luteinizing hormone (LH), with a resultant reduction in semen quality, testicular volume, penile length, and anogenital distance [34]. A cross-sectional study from Nanjing, China [103], on 664 adult men showed that seminal PFOA and PFOS levels were significantly associated with a lower percentage of progressive sperm and higher percentage of DNA fragmentation. Use of serum PFOA and PFOS levels made their association weaker compared to the levels in seminal fluids [103]. A follow-up study [33] using the same population confirmed the association between serum and semen PFOA levels with semen parameters and showed a decrease in total testosterone and free testosterone. A study in the US [69] examining semen quality among 501 male partners of couples planning pregnancy showed a correlation between PFOA and PFOS exposure and semen quality, with PFOA and PFOS being associated with a lower percentage of sperm with coiled tails. A study using 588 partners of pregnant women from Greenland, Poland, and Ukraine found that, across countries, sperm concentration, total sperm count, and semen volume were not consistently associated with PFOS or PFOA levels. The most consistent findings were inverse associations between PFOS exposure and sperm morphology [104]. A positive association between PFOA and semen motility [104] and between PFOA and sperm DNA damage [105] was not consistently found across countries. While one cannot rule out that this is a chance finding due to multiple statistical tests being performed, it is also quite likely that different mixtures of PFAS are present in differing environments, which influences the final associations with male reproductive parameters. In support of our supposition, a study utilizing 105 Danish men [37] reported that higher exposure to mixtures of PFOA and PFOS was associated with a decrease in sperm count and number of morphologically normal sperm, as opposed to a weaker association of these sperm parameters with PFOA alone. Moreover, though the highest PFOS–PFOA exposure group displayed a lower total testosterone level, it was not statistically significant. A follow-up cross-sectional study of a cohort of 247 men 5 years later by the same group [38] showed that PFOS levels were negatively associated with testosterone, but earlier findings of decreased sperm morphology were not replicated. As discussed in their publication, this discrepancy could possibly be due to a lack of highly exposed individuals, meaning that the limits of the first study would make all the individuals in the second cohort fall into the “low PFAS” category.

Vested et al., 2013 [106] examined the effects of in utero exposure to PFOA and PFOS in 169 male offspring (19–21 years of age) from a pregnancy cohort established in Aarhus, Denmark, in 1988–1989. They found trends of lower sperm concentration, sperm count, as well as higher FSH (follicle-stimulating hormone) and LH, with higher in utero exposure to PFOA. Lopez-Espinosa et al., 2016 [107] studied 1169 boys (6–9 years of age) living near a chemical plant in the Mid-Ohio Valley (US) with local contamination from PFOA in drinking water. For this cohort, a significant inverse association was found between PFOA levels and testosterone levels, and between PFOS levels with estradiol, testosterone, and IGF-1 (Insulin-like Growth Factor 1) levels. Similarly, a study with 540 Taiwanese subjects aged 12–30 years showed that serum concentrations of PFOA and PFOS were negatively associated with serum levels of sex hormone-binding globulin (SHBG), FSH, and testosterone [108].

In contrast to the above studies showing various degrees of association between PFOS/PFOA and male reproductive parameters, some other groups have found that PFAS exposure is not significantly associated with either semen quality or reproductive hormone levels [39,102,109,110]. The reason for these apparent contradictions could be: (i) composition and concentration levels of various PFAS within the exposure mixture; (ii) age of cohort; (iii) racial differences; (iv) differences in diet; and/or (v) susceptibility windows of exposure. A detailed summary table of the effects of PFOS and PFOA exposure on serum parameters and hormone levels can be found in a review by Bach et al., 2016 [3].

In conclusion, epidemiological studies point to human PFOS/PFOA exposure being associated with deleterious male reproductive outcomes and changes in hormone levels of estrogen, testosterone, LH, FSH, and SHBG. The endocrine-disrupting property of PFOS/PFOA makes associations between PFOS/PFOA levels and outcomes especially problematic, since, instead of a linear response shown by most toxic compounds, endocrine disruptors usually exhibit non-monotonic dose responses. This might, in part, be why some studies did not show dose outcomes expected of that population. It is especially interesting that the three studies [106,107,108] examining either gestational or pubertal PFAS exposure showed more consistent PFAS–testosterone associations, pointing to the exposure during developmental windows of susceptibility as being a key factor influencing reproductive outcomes. To determine the mechanism underlying the cause–effects, an examination of the results from rodent and in vitro studies is required.

### 4.3. Rodent Studies Linking PFOS and PFOA to Male Reproductive Health

The reproductive effects of PFOA have been studied in rats, mice, monkeys, and rabbits. The rat does not appear to be a suitable animal model since it shows a sex-dependent half-life of PFOA. Moreover, testosterone appears to inhibit the renal clearance of PFOA [97,99] in rats. Male and female mice exhibit an estimated half-life of 12–20 days and may be a better model system, especially for PFOA (reviewed in Post et al., 2012 [100]). Indeed, we found that, except for one study using 11–12-week-old CD rats, PFOA had no effects on reproductive hormone levels or on male reproductive health (Table 1; [29]).

PFOS and PFOA have resulted in dose-dependent developmental effects in rats and mice involving birth weight, postnatal survival, and postnatal growth in surviving animals [32,36,92,93,108,111,112,113,114,115,116]. The most common change with possible adverse effects on male reproduction on exposure to PFOS and PFOA appears to be fluctuations in hormone levels (Table 1, Table 2, Table 3 and Table 4). As described in Table 1, Table 2, Table 3 and Table 4 in more detail, for some studies, the levels of either serum or testicular testosterone appeared to decrease [32,36,92,93,108,111,112,113,114,115,116]; in others, the level of estrogen increased [36,117]. However, this was not a universal phenomenon as some studies show opposite effects, no changes in these two hormone levels [115,118,119,120], or dose-dependent changes [112]. Similarly, levels of FSH and LH displayed non-consistent effects. The receptors for hormones [121,122,123] including androgen, estrogen, and FSH were found to change following exposure. As described in Table 3 and Table 4, changes in steroidogenic enzyme levels have been reported [93,116] for PFOS exposure, which could be due to loss of Leydig cell function [30,113].

Examination of impaired spermatogenesis and reduced sperm counts has also shown variable results (refer to Table 1, Table 2, Table 3 and Table 4), with no obvious common thread linking the differences observed. The inconsistencies could be due to several factors:

(i) Lack of experimental verification of levels of PFOS and PFOA in the serum make it hard to interpret results from rodent studies. Most studies have estimated values of PFOS/PFOA based on previous studies (reviewed by Olsen et al., 2009 [129]). In most cases, PFOS/PFOA accumulation levels in the testes are ignored.
(ii) Strain differences within the tested rodent population are an added complication. Studies with endocrine disruptors have shown that some strains of rats and mice are more susceptible to endocrine disruptors than others (discussed in Ashby, 2001 [130]).
(iii) There is very little information on the food/diet fed to these rodents. It has been shown that the fatty acid content of diets could be an important source of experimental variation impacting spermatogenesis [23,131,132,133,134,135,136,137,138].

### 4.4. Challenges and Areas of Future Research

There is growing evidence to show that PFAS may act as endocrine disruptors on the reproductive system. The endocrine disruptor function of a chemical depends on its concentration within the system and can cause fluctuations in responses and non-monotonic responses, an inherent problem for both human epidemiological studies and for animal studies. Additionally, exposure levels during “windows of susceptibility”, when the organ is more susceptible to hormonal effects, may give more consistent dose outcome results, rather than at the disease endpoint. For example, the epidemiological studies [106,107,108] examining either gestational or pubertal PFAS exposure showed more consistent PFAS dose–testosterone-level associations. Similarly, PFAS exposure pre-puberty and during puberty could be an important determinant to show more consistent associations between PFAS, testosterone levels, and sperm counts or impaired spermatogenesis in rodents. However, there are many other challenges to studying PFAS-related human health effects. These include the lack of an unexposed control population, exposure to PFAS in the form of complex mixtures of variable constituents with little knowledge regarding the potential synergistic effects of mixtures of PFAS, the window of exposure susceptibility impacting PFAS-related adverse health outcomes not being known (gestational, neonatal, puberty, old age, chronic long-term), and interspecies (for rodents) and ethnic differences (humans) regarding toxicological and toxicokinetic effects of PFAS not being fully understood and further complicated by possible sex differences (reviewed by Blake and Fenton, 2020 [4]). Moreover, the reproductive health effects of long-term chronic exposure to low concentrations of PFAS on the endocrine system, including the thyroid, require further examination.

## 5. Mechanism of Action of PFOS and PFOA

PFOS and PFOA function as endocrine disruptors on the reproductive system. Animal and in vitro studies have shown that levels of testosterone decrease on increasing exposure to PFAS [32,92]. A decrease in gonadal and/or serum testosterone or increase in estrogen skews the testosterone/estrogen ratio, leading to hormonal imbalance. Aromatase is the key enzyme responsible for converting testosterone to estrogen. In support, the use of aromatase inhibitors in infertile men with low testosterone/estrogen ratios improved both hormonal and semen parameters (reviewed in Schlegel et al., 2012 [40]). Within the testis, the Leydig cells and Sertoli cells work together to sustain and regulate the development of the germ cells into the mature spermatozoa.

### 5.1. Leydig Cells

Leydig cells produce and secrete testicular testosterone, which accounts for over 95% of circulatory testosterone [35,139]. In addition to aromatase, PFAS exposure has been revealed to be significantly associated with the expression of key enzymes involved in the steroidogenesis process, thereby implying that inhibition of steroidogenic enzyme activity within Leydig cells, or a delay in the maturation/death of Leydig cells, may be a contributing factor to the effects that PFAS exert on androgen secretion in the testis. Steroidogenic acute regulatory protein (STARD1) regulates cholesterol transfer within the mitochondria, which is followed by the conversion of cholesterol to pregnenolone by CYP11A1 (cytochrome P450 family 11A1). Moreover, 17β-Hydroxysteroid dehydrogenase (HSD17B) is involved in the inter-conversion of dehydroepiandrosterone (DHEA) and androstenediol, androstenedione and testosterone, and estrone and estradiol [140]. Rodent and in vitro studies with human or rodent Sertoli cells, Leydig cells, spermatogonia, or seminiferous tubule sections have found that PFOA or PFOS decrease the levels of steroidogenic enzymes such as progesterone, STARD1, CYP11A1, HSD3B1, HSD17B, HSD11B1, and CYP17A1 [92,111,116,123,141]. Additionally, the expression levels of testicular receptors for gonadotropin, growth hormone, and IGF-1 were considerably reduced in mice exposed daily to PFOS [116].

### 5.2. Sertoli Cells

**Figure 1:**
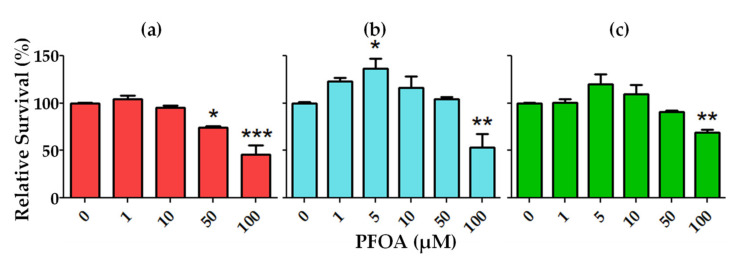
Cell survival after treatment with indicated concentrations of PFOA. Mouse Sertoli TM4 (**a**), mouse type B spermatogonia GC-1 (**b**), and mouse pre-meiotic spermatocyte GC-2 (**c**) cell lines (obtained from ATCC) were plated in triplicate in a 96-well plate at a density of 20,000 cells/well and incubated overnight. Cells were treated with PFOA at 5 different concentrations ranging from 0 µM to 100 µM followed by an incubation period of 3–4 days. Cell proliferation (MTT) assay was performed. Displayed data are from at least two individual experiments, mean ± standard error (SEM). Significance was calculated using one-way ANOVA with Dunnett’s post-test. * *p* value < 0.05, ** *p* value < 0.01, *** *p* value < 0.001.

Sertoli cells secrete androgen-binding protein in the lumen of tubules under the action of FSH [142]. They form tight junctions with each other and maintain the blood–testis barrier responsible for protecting the differentiating germ cell from the immune system. It has been shown that PFOS induces rapid disorganization of the actin- and microtubule-based cytoskeleton in primary cultures of rodent and human Sertoli cells, a phenomenon mediated by RAC-alpha serine/threonine-protein kinase (Akt1/2) [143]. As a result, the blood–testis barrier is destabilized, resulting in male reproductive dysfunction. We found that the exposure of mouse Sertoli cells to PFOA results in a dose-dependent decrease in cell survival (Figure 1a). This observation agrees with data from PFOS- and PFOA-exposed mice, where Sertoli cells showed evidence of morphologic injuries such as the presence of vacuoles, absence of Sertoli cells (“ghost” cells), and disorganization of the cell layers within the seminiferous tubules (probably due to loss of Sertoli cells). [111,127].

### 5.3. Germ Cells

Spermatogenesis is the process by which germ cells within the seminiferous tubules undergo mitotic and meiotic divisions to form the haploid spermatids, which differentiate and mature into spermatozoa and finally the mature sperm. The germ cells express androgen receptors (AR), estrogen receptor beta, as well as aromatase, and thus are constantly responding to the hormonal milieu surrounding them. Any changes in the hormone levels or receptor expression could result in impaired spermatogenesis. In male pups gestationally exposed to Bisphenol A, our laboratory experiments have shown loss of estrogen receptor beta protein in round spermatids and increased aromatase expression on the acrosomes of the developing spermatids [131,132]. A more thorough investigation of the expression of nuclear receptors estrogen receptor beta, AR, and aromatase in the PFOS/PFOA-exposed dividing, differentiating, and maturing germ cells, as well as Leydig and Sertoli cells within the seminiferous tubule, is thus lacking.

Our research (Figure 1) has found that the effects of PFOA exposure on germ cells are more complex and depend on the stage of the differentiating germ cells. In mouse spermatogonia cell lines, exposure to PFOA (Figure 1b) resulted in an increase in cell proliferation and survival at lower concentrations of PFOA and a decrease in/inhibition of cell proliferation at higher concentrations. For spermatocyte cell lines (cells at pre-meiotic stage), PFOA exposure resulted in a dose-dependent decrease in cell survival (Figure 1c). These results are worrying and require further investigation, since any perturbations in the growth and differentiation of germ cells is thought to result in dysregulation of spermatogenesis.

### 5.4. Prospective Areas of Investigation into Modes of Action

Identification of early epigenetic biomarkers for PFOS/PFOA exposure which correlate to adverse male reproductive health outcomes could result in improved monitoring of disease susceptibility in a population. Further examination is thus required into epigenetic mechanisms such as histone modifications, DNA methylation, changes in non-coding microRNA, and RNA modifications as modes of action for PFOS/PFOA in the testis. Lu et al., 2007 [144] identified differentially expressed miRNAs in mouse testes after PFOA exposure and showed that miR-133b-3p targets clathrin light chain A, which is involved in endocytosis and thus could be responsible for testicular toxicity. While PFOA and PFOS have been shown to cause global DNA methylation and differentially alter histone modifications in breast cancer cells [145,146,147], this mechanism has not been explored for cells in the testis. Recently, m(6)A demethylase ALKBH5 was shown to regulate spermatogenesis [148]. PFOA and PFOS modification of the expression of this enzyme, and of RNA epigenetics, is a fairly new field of investigation [148] which needs additional inquiry.

## 6. Looking to the Future

A more fast-track method of testing the potency of these chemicals for human male reproductive health is necessary. Organ culture/ex vivo culture, allografting human testicular fragments under the skin of sexually mature, castrated, immune-deficient mice, and germ cell transportation to rete testis of mice are some of the currently used investigative methods. A fourth method being developed is the establishment of an in vitro human testis model using human testicular cells including human Sertoli, Leydig, and germ cells, similar to mouse or rat 3-D-based testis models [149]. Going one step further, integrating these models with a high throughput method for the monitoring of environmental toxicants’ effects on male reproductive dysfunction (including sperm morphology, sperm counts, and sperm DNA integrity) would be desirable. These methods will also serve as a platform for a future mechanistic understanding of spermatogenesis.

Although the use of PFOS and PFOA has been reduced due to their health impact, the total amount of PFAS introduced into the environment has not reduced because the long-chain compounds have been replaced by short and ultra-short PFAS [53]. While short-chain PFAS potentially bioaccumulate less compared to long-chain, they are still environmentally persistent [11,150]. Their biological effects are still relatively unknown, and they are more recalcitrant to clean-up attempts. Many agencies, such as The Center for Disease Control (CDC), Agency for Toxic Substances and Disease Registry (ATSDR), the US Environmental Protection Agency (EPA), the World Health Organization (WHO), International Agency for Research on Cancer (IARC), and the National Toxicology Program (NTP), have recorded the adverse effects of PFOS and PFOA [18,19,20,77,98,151]. However, these reports only address the possible health effects associated with legacy PFAS, and there are little to no data on health outcomes associated with replacement PFAS. This needs to be rectified.

## 7. Summary

PFOS/PFOA contamination within the environment is of concern because of their long half-life in humans and their environmental persistence. Epidemiological and rodent studies suggest associations between increased PFAS exposure and changes in the levels of some key steroidogenic enzymes [92,111,116,123,141,152,153] including gonadal or serum testosterone, testicular receptors for gonadotropin, growth hormone, and IGF-1 [116] and semen parameters. The variation and inconsistencies in some effects of PFAS exposure could be explained on the basis of PFOS/PFOA being endocrine disruptors exhibiting non-monotonic responses, or animal model systems being insensitive to hormonal fluctuations. This is a recurrent problem inherent to endocrine disruptors [49] and new statistical approaches to address this issue need to applied. Moreover, examination of the epigenetic programming (miRNA, DNA methylation, and RNA and histone modifications) triggered by PFOS/PFOA during development should be considered as a highly sensitive end-point to monitor for biomarkers of exposure, because epigenetics is a key mechanism for developmental plasticity.

## Figures and Tables

**Table 1:** PFOA exposure and male fertility measures in rats.

Author	Species (Age)	Exposure	Dose	Outcome Measures	Serum Concentration
Iwabushi et al., 2017 [83]	Male Wistar rats (6–7 weeks)	Mixture of PFAS (perfluorohexanoic acid, PFOA, PFOS, perfluorononanoic acid)	1, 5, and 25 μg/L in drinking water daily for 1 month and 3 months	Hormone levels not examined. Testis not examined. Epididymal sperm counts not done.	1-month exposure PFOA: ~6.5–160 µg/kg serum volume 3-month exposure PFOA: ~8.9–184 µg/kg serum volume
Biegel et al., 1995 [29]	Male CD rats (11–12 weeks)	Ammonium PFOA (APFO)	25 mg/kg/day APFO for 14 days.	Inhibits testosterone release from Leydig cells. Testis not examined.	Not determined
Butenhoff et al., 2004 [119]	Male and female Sprague Dawley rats (6-weeks) and F1 pups at Post-natal Day (PND) 21	Ammonium PFOA (APFO)	1, 3, 10, or 30 mg/kg/day daily by gavage.	Fertility and all sperm parameters normal in all generations.	Not determined
Cui et al., 2009 [84]	Male Sprague Dawley rats (8-weeks)	PFOA	5 or 20 mg/kg/day daily for 28 days by gavage	No distinct pathological change in testes.	Whole blood 39.2 μg/mL (5 mg/kg/day); 58.8 μg/mL (20 mg/kg/day) Testicle 16.7 μg/mL (5 mg/kg/day); 16.8 μg/mL (20 mg/kg/day)
York et al., 2010 [118]	Male and female Sprague Dawley rats (6-weeks) and F1 pups at PND21	Ammonium PFOA (APFO)	0, 1, 3, 10, or 30 mg/kg/day daily by gavage.	Fertility and all sperm parameters normal in all generations.	Not determined

**Table 2:** PFOA exposure and male fertility measures in mice.

Author	Species (Age)	Exposure	Dose	Outcome Measures	Serum Concentration
Zhang et al., 2014 [111]	Male BALB/c mice (6–8 weeks)	PFOA	0.31, 1.25, 5, and 20 mg/kg/day daily for 28 days by gavage.	Reduced testosterone and progesterone in testis. Disruption of spermatogenesis in 5 and 20 mg/kg/d groups. Reduced epididymal sperm count (only 5 mg/kg/d group tested).	Testicle 5.37 μg/g (5 mg/kg/d group) and 8.06 μg/g (20 mg/kg/d group)
Liu et al., 2015 [124]	Male Kunming mice (8 weeks)	PFOA	2.5, 5, or 10 mg/kg/day daily for 14 days.	Disruption of spermatogenesis. Reduced epididymal sperm count (dose-dependent effects at all doses). Decreased expression of NRF2 (Nuclear Factor, Erythroid 2 Like 2). Inhibition of antioxidant enzymes superoxide dismutase and catalase. Upregulation of p-p53 and BAX (BCL2 Associated X, Apoptosis Regulator) expression and downregulation of BCL-2 (B-Cell CLL/Lymphoma 2) expression in testis.	Not determined
Lu et al., 2016 [125]	Male BABL/c mice (6–8 weeks)	PFOA	1.25, 5, or 20 mg/kg/day daily for 28 days by gavage.	Disruption of blood–testes barrier and immune privilege observed in all three PFOA groups.	Not determined
Song et al., 2018 [115]	Female pregnant mice	PFOA	1, 2.5, or 5 mg/kg/day daily by gavage	Reduced level of testosterone in male offspring on PND 21. Dose-dependent damage to testis. Number of Leydig cells decreased in 2.5 and 5 mg/kg PFOA groups on PND 21 and 70.	Not determined

**Table 3:** PFOS exposure and male fertility measures in rats.

Author	Species (Age)	Exposure	Dose	Outcome Measures	Serum Concentration
Iwabushi et al., 2017 [83]	Male Wistar rats (6–7 weeks)	Mixture of PFAS (C6A, PFOA, PFOS, C9A)	1, 5, and 25 μg/L in drinking water daily for 1 month and 3 months	Hormone levels not examined. Testis not examined. Epididymal sperm counts not done.	1-month exposure PFOS: 1.09–17.2 µg/kg serum volume 3-month exposure PFOS: 2.7–73.7 µg/kg serum volume
Cui et al., 2009 [84]	Male Sprague Dawley rats (8 weeks)	PFOS	5 or 20 mg/kg/day daily for 28 days by gavage	No distinct pathological change in testes	Whole blood 72.0 μg/mL (5 mg/kg/day); not determined (20 mg/kg/day), all died within 26 days exposure. Testicle 39.5 μg/mL (5 mg/kg/day); 127 μg/mL (5 mg/kg/day)
López-Doval et al., 2014 [114]	Male Sprague Dawley rats (8 weeks)	PFOS	0.5; 1.0; 3.0; and 6.0 mg/kg/day for 28 days by gavage	Circulating levels of LH and testosterone decrease and FSH increase. Disrupts male reproductive axis activity.	Not determined
Zhao et al., 2014 [32]	Female pregnant Sprague-Dawley rats	PFOS	5, 20 mg/kg/day from gestational day 11–19 by gavage.	Male F1 generation examined at gestational day 20. Decreased testosterone, impaired fetal Leydig cells with reduced number. Decreased expression of genes expressed by Leydig cells including Cyp11A1 and decreased cholesterol levels.	Not determined
López-Doval et al., 2016 [122]	Male Sprague Dawley rats (8 weeks)	PFOS	1.0; 3.0; and 6.0 mg/kg/d daily for 28 days by gavage	PFOS inhibits both gene and protein expression of FSH receptor and AR at testicular level. Testis not examined.	Not determined
Li et al., 2018 [113]	Male Sprague Dawley rats (PND35)	PFOS	5 or 10 mg/kg/day starting PND 35 for 21 days by gavage	Lowered serum testosterone levels, decreased expression of genes expressed by Leydig cells (Lhcgr (LH/choriogonadotropin receptor), Cyp11a1, and Cyp17a1).	Not determined
Li et al., 2018 [113]	Male Sprague Dawley rats (4 weeks)	PFOS	5 or 10 mg/kg/day starting on PND 35 for 21 days by gavage	5–10 mg/kg/d reduced epididymal sperm count and serum testosterone levels. Promoted immature Leydig cell apoptosis. 10 mg/kg/d disrupted Leydig cell specific gene expression (LHCGR, CYP11A1, and CYP17A1). Delayed Leydig cell development during puberty.	Not determined
Zhang et al., 2020 [93]	Female pregnant Sprague-Dawley rats	PFOS	1 or 5 mg/kg/day from gestational day 5–20 by gavage.	Male F1 generation examined at PND1, 35, 90. Decreased serum testosterone levels. Decreased levels of Scarb1 (Scavenger receptor class B type 1), Cyp11a1, Cyp17a1, and Hsd17b3, Dhh (Desert hedgehog homolog), and Sox9 (SRY-related HMG-box). Inhibition of Leydig cell proliferation.	Not determined
Luebker et al., 2005 [120,126]	Male-female Sprague Dawley rats (6-weeks) and F1 pups	PFOS	0.1, 0.4, 1.6, and 3.2 mg/kg/day throughout the 2-generational study	Fertility and all sperm parameters normal in all generations. Developmental mortality observed for 1.6 and 3.2 mg/kg/day groups.	Not determined

**Table 4:** PFOS exposure and male fertility measures in mice.

Author	Species (Age)	Exposure	Dose	Outcome Measures	Serum Concentration
Wan et al., 2011 [116]	CD1 male mice (8 weeks)	PFOS	1, 5, or 10 mg/kg/day for 7, 14, or 21 days by gavage.	For 10 mg/kg/d, day21: Serum testosterone levels decreased. Epididymal sperm counts decreased. mRNA expression levels of steroidogenic enzymes (i.e., StAR, CYP11A1, CYP17A1, 3beta-HSD, and 17beta-HSD) were reduced.	Not determined
Qiu et al., 2013 [127]	Male ICR mice (8 weeks)	PFOS	0.25, 2.5, 25, and 50 mg/kg/day for 28 days by gavage.	For ≥ 2.5mg/kg/day: Significant dose-dependent decrease in sperm count. Significant increase in Sertoli cell vacuolization and disruption of spermatogenesis, significant loss in blood–testis barrier.	Not determined
Qiu et al., 2016 [128]	Male ICR mice (8 weeks)	PFOS	0.5, 5, and 10 mg/kg/bw daily for 4 weeks by gavage	Dose-dependent decrease in sperm count. Loss in blood–testis barrier at dose of 5 mg/kg/bw and higher	Not determined
Qu et al., 2016 [121]	Male C57 mice (6–8 weeks)	PFOS	0.5 and 10 mg/kg/day for 5 weeks by gavage	Serum testosterone levels decreased. Higher incidence of apoptotic cells, and vacuolations observed in spermatogonia, spermatocytes, and Leydig cells for 10 mg/kg/day group.	Not determined
Lai et al., 2017 [112]	Female pregnant CD-1 mice (6−8 weeks)	PFOS	0.3 or 3 mg/kg/day	On PND1, aberration of lipid metabolism, oxidative stress and cell-junction signaling, perturbations of lipid mediators. On PND63 reduction in serum testosterone and epididymal sperm count	PFOS in PND1 testis: ~0.6 µg/g (0.3 mg/kg/d group), ~3.8 µg/g (3 mg/kg/d group)

## Data Availability

No Additional Data to report.
